# Obesity, organ failure, and transplantation: a review of the role of metabolic and bariatric surgery in transplant candidates and recipients

**DOI:** 10.1007/s00464-024-10930-8

**Published:** 2024-07-01

**Authors:** Omar M. Ghanem, Alejandro Pita, Mustafa Nazzal, Shaneeta Johnson, Tayyab Diwan, Nabeel R. Obeid, Kristopher P. Croome, Robert Lim, Cristiano Quintini, Bryan A. Whitson, Holly Ann Burt, Charles Miller, Matthew Kroh, SAGES & ASTS

**Affiliations:** 1 Department of Surgery, Mayo Clinic Rochester, Rochester, MN USA; 2 Department of Surgery, Cleveland Clinic Foundation, Cleveland, OH USA; 3 Department of Surgery, Saint Louis University Hospital, St. Louis, MO USA; 4 Department of Surgery, Morehouse School of Medicine, Atlanta, GA USA; 5 Department of Surgery, University of Michigan, Ann Arbor, MI USA; 6 Department of Transplant, Mayo Clinic Florida, Jacksonville, FL USA; 7 Atrium Health Carolinas Medical Center, Wake Forest University School of Medicine, Charlotte, NC USA; 8 Digestive Disease Institute, Cleveland Clinic Abu Dhabi, Abu Dhabi, United Arab Emirates; 9 Division of Cardiac Surgery, Department of Surgery, The Ohio State University Wexner Medical Center, Columbus, OH USA; 10 Society of American Gastrointestinal and Endoscopic Surgeons (SAGES), Los Angeles, CA USA

**Keywords:** Obesity, Solid organ transplant, Bariatric surgery, Liver transplant, Kidney transplant

## Abstract

Obesity is a risk factor for kidney, liver, heart, and pulmonary diseases, as well as failure. Solid organ transplantation remains the definitive treatment for the end-stage presentation of these diseases. Among many criteria for organ transplant, efficient management of obesity is required for patients to acquire transplant eligibility. End-stage organ failure and obesity are 2 complex pathologies that are often entwined. Metabolic and bariatric surgery before, during, or after organ transplant has been studied to determine the long-term effect of bariatric surgery on transplant outcomes. In this review, a multidisciplinary group of surgeons from the Society of American Gastrointestinal and Endoscopic Surgeons and the American Society for Transplant Surgery presents the current published literature on metabolic and bariatric surgery as a therapeutic option for patients with obesity awaiting solid organ transplantation. This manuscript details the most recent recommendations, pharmacologic considerations, and psychological considerations for this specific cohort of patients. Since level one evidence is not available on many of the topics covered by this review, expert opinion was implemented in several instances. Additional high-quality research in this area will allow for better recommendations and, therefore, treatment strategies for these complex patients.

**Supplementary Information:**

The online version contains supplementary material available at 10.1007/s00464-024-10930-8.

## Introduction

According to the United Network of Organ Sharing, about 42 800 organ transplants were performed in 2022, including 25 000 kidney, 9500 liver, 4100 heart, and 2700 lung transplantation surgeries [1]. There are currently about 103 555 patients in need of organ transplantation, with 58 635 patients on the active waiting list [2]. Each month, about 1500 new patients are added to the transplant list, whereas 900 are deactivated from the list for varying reasons, including death [2]. Obesity is a main limiting factor for patients seeking transplantation because several studies have concluded that organ transplant outcomes are worse in patients with a body mass index (BMI) of > 30 kg/m^2^ compared with individuals with lower BMIs [3, 4]. Additionally, the prevalence of obesity in the US and worldwide has reached epidemic proportions. Currently, 40% of the adult population in the USA is living with obesity [5]. This, in turn, raises the prevalence of patients with obesity requiring solid organ transplantation (SOT).

Additionally, weight gain is expected within the first years after SOT, with a proportional increase in the recipient’s rate of cardiovascular events and long-term mortality [6, 7]. Given that transplant patients will be started on medications, including steroids, there may be some reluctance to refer these patients for metabolic and bariatric surgery (MBS) because of the risks of staple-line leaks and marginal ulceration after surgery [8]. Obesity medicine specialists and MBS surgeons may be hesitant to treat transplant patients for fear of malnutrition and malabsorption and compromising transplant outcomes. A review of the Metabolic and Bariatric Surgery Accreditation and Quality Improvement Program database shows the 30-day major complication rate to be 3 times higher in transplant patients, although mortality was similar to the general population [8]. Other studies, however, show promising results for offering MBS after transplantation. Fang et al [9] compared patients who underwent MBS prior to transplant against patients who underwent MBS after transplant. This study showed that patient survival and graft survival were equivalent, as were the weight loss and total rate of postoperative complications [9]. Other studies have shown favorable medium- and long-term weight loss outcomes and diabetes resolution in patients with immunosuppression, including steroids [10, 11]. Overall, MBS done prior to transplant or simultaneously with the transplant seems to prevent weight gain often seen after transplant.

The relationship between obesity and transplant surgery remains a complex one. There is a scarcity of high-quality studies reporting data on long-term follow-up after MBS in patients who underwent organ transplants. Another aspect of MBS to evaluate is the successful treatment of obesity-related medical conditions such as chronic obstructive pulmonary disease, type-2 diabetes mellitus (T2DM), hypertension, hyperlipidemia, and obstructive sleep apnea. Although weight loss from MBS appears beneficial in transplant recipients, reluctance among physicians to refer transplant candidates with obesity to undergo bariatric surgery is still observed.

### The Society of American Gastrointestinal and Endoscopic Surgeons/The American Society for Transplant Surgery

The American Society for Transplant Surgery (ASTS) is a nonprofit professional society founded in 1978. It consists of surgeons trained in organ transplantation with a mission to promote and encourage education and research with respect to transplantation surgery. The Society of American Gastrointestinal and Endoscopic Surgeons (SAGES) is a nonprofit professional society that was founded in 1980 with an initial focus on laparoscopic and endoscopic surgery. As minimally invasive techniques have reached many different disciplines, SAGES consists of many surgical subspecialists, including metabolic and bariatric surgeons. The improvement in minimally invasive MBS procedures led to a better acceptance of bariatric surgery to treat patients with obesity. The ASTS and SAGES have agreed to collaborate on this manuscript to help provide data on the controversies surrounding obesity and transplantation surgery. The overall purpose of this manuscript is to help determine the best practices for those patients who would benefit from both a transplant and MBS and to provide guidance for physicians who treat these patients. This manuscript summarizes the current existing evidence available in the literature and has been reviewed by expert surgeons from SAGES and ASTS. It is not intended to serve as a guideline or an official statement from either society.

## Materials and methods

An extensive literature review was coordinated and performed by the authors of this manuscript after defining research questions and relevant keywords. The authors coordinated the different tasks, which included developing the review protocol, describing inclusion and exclusion criteria, selecting an adequate search strategy and search engines, ensuring quality assessments, and extracting the data for final synthesis. Using selected databases such as PubMed, Science Direct, and Springer Link, several keywords were used to locate relevant articles. The keywords included “Obesity,” “Metabolic Surgery,” “Bariatric Surgery,” “Organ Transplantation,” “Kidney Transplant,” “Liver Transplant,” “Heart Transplant,” “Lung Transplant,” and “Weight Loss.” The final article inclusion was approved by all the authors who implemented expert opinion to synthesize the collected information and made scientific suggestions where literature leaves off and gaps in knowledge persist (Supplementary Table).

### Obesity as a risk factor for organ failure

Obesity is associated with the development of adipose tissue inflammation, resulting in insulin resistance, metabolic dysfunction, and the development of multiple chronic diseases, including end-stage kidney, liver, heart, and lung disease, and eventually organ failure [12].

Multiple studies demonstrate that obesity in patients with chronic kidney disease (CKD) induces glomerular hyperfiltration, which eventually leads to structural abnormalities in the glomeruli in a manner analogous to that described in reduced renal mass states [13]. From the bariatric perspective, about 21% of patients with CKD have obesity [14]. An estimated 750 MBS are performed on patients with CKD annually and 400 to 450 on those with end-stage kidney disease (ESKD) [15, 16]. These numbers suggest that only a small number of these patients are being referred to or undergoing this surgery. Nonetheless, MBS is utilized in patients with CKD and ESKD to make them eligible candidates for transplantation. Additionally, some of the risk factors for the development and progression of CKD are related to obesity itself, namely hypertension and T2DM [16]. As such, it may be possible that earlier utilization of MBS and efficient obesity management in patients with CKD prevent the progression to ESKD.

The effect of obesity is quite different for patients in need of liver transplantation (LTx), given that cirrhosis from metabolic dysfunction-associated steatotic liver disease (MASLD) is one of the most common indications for liver transplant [17]. MASLD is a disease of insulin resistance that becomes more pronounced in patients with obesity. As MASLD progresses to metabolic dysfunction-associated steatohepatitis (MASH), which can lead to cirrhosis, it is evident that obesity is a main risk factor and reason for patients to eventually require LTx [17]. In a recent report, 23% of liver transplant candidates have class I obesity, 10% have class II, and 4% have class III [18]. Multiple studies, including the Surgical Procedures and Long-term Effectiveness in NASH Disease and Obesity Risk trial, demonstrated a significant decrease in adverse liver outcomes in patients who underwent MBS to reduce their BMI prior to transplant [19]. As such, some have advocated for MBS surgery to be performed simultaneously with LTx to optimize surgical outcomes [20].

Obesity is also implicated in the progression of heart disease and failure through multiple mechanisms. The increased intra-abdominal pressure can cause changes in cardiac hemodynamics, structure, function, and conduction, which eventually lead to obesity-related cardiomyopathy [21]. Also, the metabolic changes involving insulin resistance and cardiac lipotoxicity trigger adipokine release, inflammation, and endothelial dysfunction, which further aggravate the circulatory and cardiac systems [21]. Lastly, the accumulation of fat in the mediastinum and the abdominal cavities in patients with preexisting lung disease has been shown to significantly alter lung and chest wall function [22]. The increased weight can also cause decreased lung compliance with a reduction in airway size and tone, ultimately leading to the progression to lung failure [23].

### MBS optimization considerations in transplant patients

MBS may be indicated and utilized in pretransplant and posttransplant clinical settings. Although indications for use may differ in these patient populations, both require significant optimization considerations to ensure optimal outcomes and an optimized patient safety profile.

#### Optimization of pretransplant patients for MBS

Pretransplant patients presenting for MBS typically have higher preoperative, intraoperative, and postoperative risks and significant optimization considerations compared with the general MBS population [24]. Preoperative risks may include hypertension, T2DM, and cardiac disease in patients with ESKD; cardiac and pulmonary risks in cardiac transplant candidates; pulmonary hypertension in lung transplant candidates; and coagulation abnormalities in liver transplant candidates [24]. These risks increase anesthesia and perioperative risks and require vigilance with preoperative evaluation. MBS may be an option for patients with compensated cirrhosis; however, it is often too high-risk of patients with decompensated liver disease [7]. Despite some studies supporting favorable perioperative outcomes of simultaneous liver transplant surgery and MBS, there remains an increased operative and postoperative risk due to the complexity of the combined procedure [25]. Patients with end-stage cardiopulmonary failure present a challenging preoperative dilemma. The time-sensitive nature of their disease process may rule out the possibility of meaningful weight loss to qualify for transplantation. Therefore, a comprehensive preoperative evaluation should be undertaken and include imaging, tests, and consultation with specialists to ensure patient optimization [24].

Another important patient-related aspect to evaluate prior to MBS and SOT is the patient’s frailty. In fact, frailty has been correlated with worse overall surgical outcomes in pretransplant and posttransplant settings, particularly for patients with other risk factors such as obesity. Additionally, patients undergoing significant weight loss surgery are at an increased risk of aggravating frailty or becoming frail if they were nonfrail at baseline, which might increase the morbidity and mortality associated with this combined approach of MBS and SOT. Therefore, careful preoperative risk stratification is warranted for this specific patient population, coupled with a close multidisciplinary follow-up after surgery to minimize morbidity and mortality [26, 27].

Lastly, patients who undergo hypoabsorptive bariatric procedures are at increased risk of long-term malnutrition and nutritional deficiencies, which might affect graft function and survival. Careful preoperative assessments by a multidisciplinary team should include a range of metabolic tests for serum vitamins, iron studies, serum albumin, and prealbumin levels. Identifying micronutrient and macronutrient deficiencies early after surgery and administering proper supplementation will decrease the morbidity and mortality associated with combined MBS and organ transplantation [28].

#### Optimization of posttransplant patients for MBS

The prevalence of obesity in patients who underwent SOT is significant, with studies reporting that 1 in 3 patients develop obesity within the first 3 years of transplantation due to immunosuppressive medications and other mechanisms [29, 30]. MBS has been shown to have a similar successful percentage of excess weight loss in posttransplant and nontransplant patients [30]. Additionally, posttransplant patients who undergo MBS are demonstrated to have a potential improvement in graft function and decreased graft steatosis and fibrosis in liver transplant and renal transplant patients [31]. However, patients with previous transplants have also been demonstrated to have a higher incidence of intraoperative complications such as increased adhesions, hemorrhage, and longer operative times, as well as higher postoperative complications and readmission rates [31, 32].

The management of medication and treatment regimens may also pose significant challenges in the posttransplant period. Immunosuppressive medications may impair healing and increase the risk of surgical or other infections [33]. This immunosuppression also increases the risk of developing staple-line leaks and subsequent poor healing. Intraoperatively, hypotension, hypertension, and other intraoperative insults may impact the transplanted organ negatively [33]. Optimization and careful management of the transplant patient are imperative to prevent such insults and the associated sequelae within the perioperative and immediate postoperative period.

#### Timing of MBS and SOT

**Fig. 1:**
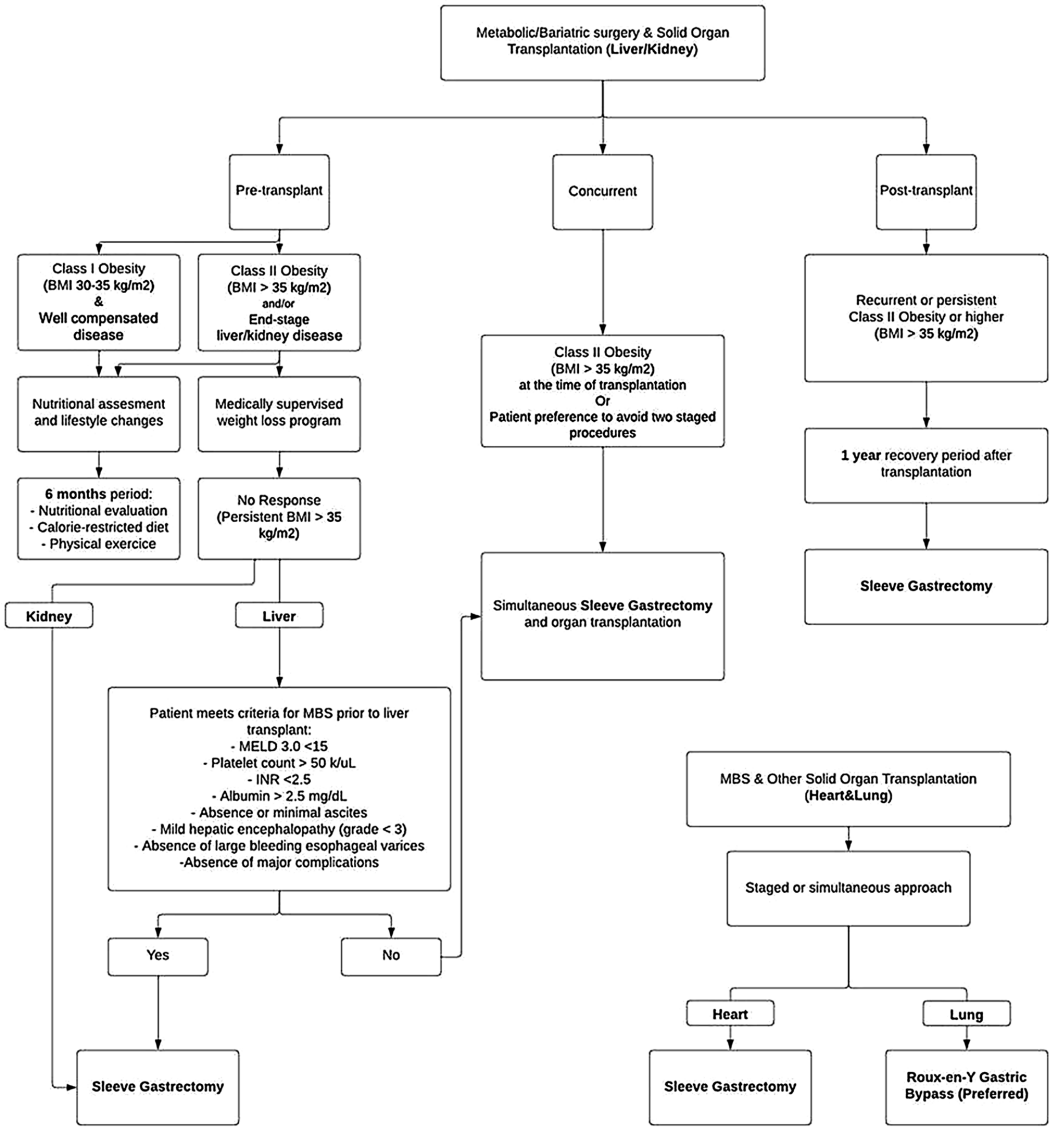
Consort diagram. BMI, body mass index; MELD, model for end-stage liver disease

MBS prior to SOT has been the focus of much scholarly inquiry in recent years; however, concurrent surgery— particularly sleeve gastrectomy (SG) at the time of transplantation—has been implemented at some centers for specific indications with early success (Fig. 1). MBS has been offered to posttransplant patients with significant health benefits and a favorable safety profile during active immunosuppression [10]. In this section, the current indications for MBS among transplant patients will be reviewed. The different timings of MBS surgery, including pretransplant, concurrent surgery, and posttransplant, will be discussed as they pertain to different transplanted organs. Finally, a review of associated clinical outcomes and trends in practice management is presented.

### LTx

In recently proposed updated guidelines to the National Institutes of Health recommendations for MBS in the general population, indications now advocate MBS surgery in patients with a BMI of > 35 kg/m^2^, regardless of the presence or absence of comorbidities [34]. However, these guidelines have not been fully adopted yet, particularly by insurance companies. When considering MBS in the liver transplant patient population, the process of patient selection, timing of procedure, and goal of treatment should focus on obtaining optimal outcomes not only in terms of weight loss and comorbidity mitigation but also in regard to transplantation. As such, the goals of the MBS procedure may differ from those of the general patient population, and the definition of a good outcome includes the optimization of long-term results following LTx. MBS has been proven to be safe and effective in this patient population when performed in centers with ample experience in both MBS and LTx. Although different MBS approaches have been reported in patients with end-stage liver disease (ELD), SG is likely the procedure of choice [29]. It is technically less demanding, does not require additional bowel anastomoses, preserves the natural anatomy favorable for biliary access through endoscopic retrograde cholangiopancreatography, and provides the least possible interference with the pharmacokinetics of immunosuppressive medications while achieving adequate metabolic results [35].

#### MBS before liver transplant

Select patients with well-compensated ELD can be considered candidates for MBS prior to LTx. When patient selection is appropriate, the risk of major postbariatric surgery complications, including major adverse liver-related outcomes, is approximately 2% to 5% [19, 36]. As such, MBS can be safely performed in selected patients who are in the process of being evaluated for or already waitlisted for LTx. In patients with advanced chronic liver disease and at least class I obesity (BMI > 30 kg/m^2^; corrected for fluid retention), the European Association for the Study of the Liver guidelines recommend nutritional assessment and lifestyle changes, including consideration of screening for sarcopenic obesity [37]. Patients with ELD with BMI of > 35 kg/m^2^, particularly those with manifestations of metabolic syndrome and comorbid conditions, could benefit from a referral for a medically supervised weight loss program. All patients being considered may be enrolled in a lifestyle modification program for a period of at least 6 months, involving an in-depth nutritional evaluation, calorie-restricted diet, and physical exercise program. Patients who fail to respond to lifestyle modification (persistent BMI > 35 kg/m^2^ in the absence of clinically significant fluid overload) could be considered for SG prior to LTx. The American Gastroenterology Association clinical practice guidelines recommend assessment for clinically significant portal hypertension (hepatic venous pressure gradient > 10 mmHg or clinical evidence of portal hypertension on imaging and endoscopy) for all patients with cirrhosis being considered for MBS, as well as concurrent candidacy evaluation for potential LTx [38].

When defining candidacy for MBS prior to LTx, the following criteria may be considered for inclusion: model for ELD (MELD) score 3.0 < 15, platelet count of > 50 k/uL, international normalized ratio of < 2.5, albumin levels of > 2.5 mg/dL, absence or minimal presence of ascites (including patients well-controlled with diuretics or transjugular intrahepatic portosystemic shunt), no more than mild hepatic encephalopathy (grade < 3), and nothing more than small, nonbleeding esophageal varices [35–37].We suggest that pretransplant MBS be avoided in patients with evidence of clinically significant malnutrition, hepatopulmonary syndrome, portopulmonary hypertension, and moderate-to-severe cardiac comorbid conditions. In patients with evidence of clinically significant portal hypertension, including large perigastric or esophageal varices, severe gastric antral vascular ectasia, or those where large collaterals or splenorenal shunts in close proximity to the gastric fundus are present, it may be preferred to perform bariatric surgery and LTx concurrently [39–41].

#### Simultaneous MBS and liver transplant

In addition to patients with significant portal hypertension and the aforementioned varices, patients with a BMI of > 35 kg/m^2^ at the time of LTx may be considered for simultaneous SG. However, BMI criteria may be affected by the presence of clinically significant fluid overload often found in these patients (including ascites, lower extremity edema, and anasarca); thus, a detailed and comprehensive assessment of the physical condition and nutritional status of the patient must be performed. Patients who could be considered for the simultaneous approach may include those who failed to sustain weight loss prior to LTx with lifestyle modification alone and patients who declined MBS prior to transplant due to the risk of decompensation or preference to avoid 2 separate operations. In all cases, we suggest the SG be performed after implantation of the allograft has been completed. Simultaneous MBS and LTx have been safely performed in patients within the entire MELD score spectrum; therefore, elevated MELD score alone may not be considered a contraindication to the combined approach [42]. In cases of hemodynamic instability or other intraoperative complications, a brief-interval staged approach (LTx followed by completion of biliary reconstruction with SG in the ensuing days) can be considered [43]. The need for simultaneous transplantation of another organ or combined procedure (such as kidney transplantation [KTx], coronary artery bypass grafting, and cardiac valve replacement) may not be considered an absolute contraindication to a combined approach; however, extreme caution must be exercised as the underlying clinical conditions of these patients may put them at increased risk of complications, and there is currently a paucity of literature in support or against this practice.

#### MBS after liver transplant

MBS after LTx may be reserved for patients with recurrent or persistent class II obesity (BMI > 35 kg/m^2^) or higher, particularly those who have obesity-related medical conditions. A recovery period of approximately 1 year is suggested following LTx before undergoing MBS because there are substantial physiologic adjustments and potential complications that may arise in the early posttransplant course [40]. At the time of MBS, an increased level of technical difficulty may occur as a result of adhesions, and this procedure should be performed in a center with expertise in both MBS and LTx. Careful review of previous operative reports and imaging studies by a team consisting of both a metabolic/bariatric surgeon and a transplant surgeon is paramount to ensure anatomy is well understood. It is often advantageous for the bariatric surgery team to collaborate with a transplant surgeon during the MBS procedure. Following these principles, sustained adequate results can be achieved while minimizing risk in this patient population.

### KTx

KTx remains the definitive treatment for patients with ESKD. A main concern regarding KTx is the associated risk of short- and long-term complications. Patients with obesity have an even higher risk compared with patients without obesity. Among the available surgical approaches for KTx, robotic-assisted transplantation has shown a decrease in obesity-related complications and the risks of graft loss [44]. These potential complications include increased risk of delayed graft function, acute rejection, posttransplant diabetes, suboptimal perioperative outcomes (eg, degree of technical difficulty, operative time, hospital length of stay, and wound complications), graft loss, and mortality. As a result, transplant programs have established relative and absolute contraindications based on the degree of obesity in potential transplant candidates, usually ranging from a BMI greater than 35 to 40 kg/m^2^. Moreover, a direct correlation between higher BMI and difficulty of access to organ transplants with longer waiting times has been established, which justifies optimal weight loss for patients on the transplant list [45]. Recent studies evaluating the effect of pharmacologic treatment (glucagon-like peptide 1 receptor agonists) in promoting weight loss for patients with ESKD have shown a modest glycemic and weight benefit while being associated with gastrointestinal side effects that might limit adherence [46]. Although pharmacologic treatment has been increasingly addressed in recent literature, minimally invasive SG is often still considered the preferred approach [47]. Among bariatric procedures, the SG has been associated with lower complication rates compared with Roux-en-Y gastric bypass (RYGB) while achieving superior weight loss and transplant results. A specific concern of RYGB in patients awaiting kidney transplants is the increased risk of oxalate stone formation, which might affect graft survival. RYGB portends up to a threefold increase in calcium oxalate stone formation postoperatively, and medical providers should be cognizant of this complication when referring patients to MBS [48].

#### MBS before kidney transplant

Patients with obesity (BMI > 30 kg/m^2^) and CKD may be enrolled in a medically supervised, multidisciplinary weight management program focused on lifestyle modification, including an in-depth nutritional assessment, calorie-restricted diet, and physical exercise plan. Patients with class II obesity and beyond, diagnosed with ESKD or CKD with a glomerular filtration rate of < 20 mL/min, could benefit from inclusion in an MBS program, starting with a lifestyle modification program and consideration for SG if sustained weight loss is not achieved (persistent BMI > 35 kg/m^2^ in the absence of clinically significant fluid overload) [49]. Given the potential need for kidney transplants in this patient population, MBS should be conducted under a transplant bariatric program or in centers with expertise in both MBS and renal transplantation. Patients with CKD and a glomerular filtration rate of > 20 mL/min meeting similar criteria can also be considered for MBS, although a transplant bariatric program should not be a requirement in these cases [49]. In this specific cohort of patients with ESKD and obesity, SG, unless contraindicated, emerges as the preferred procedure compared with RYGB. A recent meta-analysis comparing the outcomes of patients who underwent MBS with or without a diagnosis of ESKD demonstrated that patients with ESKD had a lower mortality rate, complication rate, and hospital stay following SG compared with RYGB. Nevertheless, patients with ESKD also presented higher overall morbidity compared with patients without ESKD who underwent MBS, justifying the need for postoperative close monitoring [50].

#### Simultaneous MBS and kidney transplant

Patients who fail to maintain sustained weight loss with a BMI in the 35 to 40 kg/m^2^ range or have a preference to avoid 2 separate procedures, may be eligible for simultaneous SG and KTx [5]. In the case of waitlisted patients where a living donor is available, the simultaneous approach would be preferred, in order to avoid unnecessary prolongation of the pretransplant course. A recent meta-analysis by Fernando et al [51] showed the simultaneous approach to be safe and effective in terms of weight loss and renal function improvement without compromising the renal graft function or increasing postoperative complication rates.

#### MBS after kidney transplant

Given the obesity-associated adverse outcomes in kidney transplant recipients, posttransplant MBS has been associated with improved long-term results and a decreased incidence of transplant-related complications. Kidney transplant recipients with class II obesity and beyond might benefit from an SG, regardless of the presence of comorbidities [49]. Performing the operation within the scope of a transplant bariatric program or at least in a center with expertise in both MBS and renal transplantation is recommended, as the postoperative care of these patients, particularly immunosuppression management, differs from the general postoperative bariatric practice. In this specific cohort of patients, there remains a concern regarding the effect of immunosuppressant medications on postoperative complication rates. Nevertheless, studies evaluating the rates of specific complications, particularly leaks, identified a statistically similar occurrence regardless of immunosuppression status in patients who underwent kidney transplants followed by MBS [50]. We suggest that MBS should be avoided in the first year following transplantation to reduce the risk of transplant-related complications and to protect the transplant outcomes of the program.

### Heart transplantation

Obesity exhibits complex and diverse effects on the cardiovascular system, leading to an increased risk of heart failure (HF) compared with the general population [52]. Cardiac transplantation for eligible candidates remains the standard of care for definite end-stage HF management [52]. Unfortunately, the worldwide shortage of organ transplants is reflected by long listing durations and low rates of heart transplantations [53]. As a temporary measure, surgical implantation of a left ventricular assist device (LVAD) for selected patients may be recommended as a bridging therapy prior to transplantation [54]. Given the effectiveness of recent LVAD models and the survival benefits they confer, stable patients with an implanted LVAD are no longer given top priority on the transplant list since 2018 [55]. Patients with obesity and LVADs are at increased risk of morbidity [56]. Additionally, the International Society for Heart and Lung Transplantation criteria for eligibility recommendations have also been updated in 2016, with the addition of a threshold BMI of > 35 kg/m^2^ as a relative contraindication to heart transplant [53]. Despite patients with obesity and end-stage HF being encouraged to lose weight, only a minority of these patients successfully achieve the required weight loss, in part due to their limited heart function and restricted exercise tolerance [57]. Patients with increased amounts of epicardial adipose tissue have a more accelerated time course in the progression of their HF and symptoms [58]. In addition, contrary to the early observations of significant weight loss with the HeartMate XVE, patients with the subsequent LVAD models have demonstrated a paradoxical long-term weight gain after implantation partially due to the improved functional status, increased appetite, and metabolic shifts toward anabolism [59, 60]. Therefore, achievement of optimal BMI using a combined approach of MBS and LVAD implantation could be considered as a bridge prior to cardiac transplantation [61, 62].

A recent meta-analysis by Challapalli et al. [52] comparing the outcomes of staged vs simultaneous MBS and cardiac transplantation in a limited series of 59 patients found no significant differences in terms of long-term outcomes, postoperative complication rates, or overall survival between both surgical approaches. Procedure safety remains an important concern for patients undergoing this combined surgery [63]. Reported mortality rates after 30 days of surgery are nonnegligible, with 1-year mortality reaching up to 15%, which is considerably higher than the 0.11% to 0.23% mortality rates after MBS alone [64]. Nevertheless, these rates are still low compared with the 40% to 50% 1-year mortality of nonsurgical patients with LVAD and obesity [65]. Prevalent complications to be aware of in this specific cohort of patients include major adverse [66]. cardiovascular events, thrombosis, and GI-related bleeding that affects almost one-fifth of patients Newer LVAD models such as the HeartMate 3 increase hemocompatibility and allow for lower anticoagulation and antiplatelet use perioperatively, with likely lowers the bleeding risk [53].

Recent studies demonstrate that 60% to 75% of patients who undergo combined MBS and LVAD implantation achieve the BMI requirement for a listing of < 35 kg/m^2^ within the first year (8–14 months) [67]. In addition, 35% to 45% of patients are successfully transplanted after MBS with a significantly decreased need for diuretics, vasodilators, and anticoagulant medication compared with nonsurgical patients [68, 69]. These results suggest a potential role for MBS in select cases of severe end-stage HF management.

SG is typically the most appealing option [70], given its significant weight loss outcomes, short operative duration, and low-technical complexity. It is also important to evaluate the duration from MBS to heart transplantation, which is mainly driven by the time to reach optimal weight. Doubling the amount of time spent on the waiting list was associated with a 10% increase in the odds of graft failure within 1 year after transplant [71]. This brings into question the possibility of exploring the addition of antiobesity medication as an adjunct therapy for these patients to hasten the time to transplant.

### Lung transplantation

The latest Pulmonary Transplantation Council of the International Society for Heart and Lung Transplantation criteria of eligibility included a BMI of ≥ 35 kg/m^2^ as a relative contraindication for lung transplantation [72]. Long-term studies have confirmed that patients at the extremes of obesity have significantly higher rates of chronic lung allograft dysfunction and mortality [73–75].In patients with obesity, the higher BMI risk is attributed to hypoventilation and microaspiration [76]. The selection of an appropriate MBS procedure for patients awaiting lung transplants continues to be a subject of discussion.

Limited research exists regarding MBS specifically in lung transplant patients. Preliminary studies suggest that these procedures may be both safe and advantageous. In a recent systematic analysis of 3 studies involving 28 lung transplant patients [77], demonstrated that RYGB was the most frequently performed procedure, accounting for 64.2% of cases, followed by SG at 32.1%. In one of the included studies, significant improvements in median BMI were observed at the 1-year follow-up, resulting in a total weight loss of 22% [78]. This follow-up also demonstrated significant improvements in pulmonary function tests. Importantly, there were no reported cases of mortality, and out of 7 potential lung transplant candidates, 6 became eligible for transplantation after undergoing MBS. Additionally, 3 patients experienced improvements in pulmonary function tests and quality of life to the extent that lung transplantation was no longer indicated.

Laparoscopic approaches are generally preferred due to their minimally invasive nature and shorter recovery times. In the single-center experience by Ardila-Gatas et al. [78], 25 patients with interstitial lung disease underwent MBS, with 17 patients undergoing RYGB and 7 patients undergoing SG. The decision on which procedure to pursue varied among patients based on various factors, with some patients expressing a preference in some cases. In certain cases, SG was chosen due to the elevated surgical risks associated with RYGB, whereas a subset of patients was deliberately assigned RYGB to mitigate the increased risks of developing gastroesophageal reflux disease (GERD) after SG because this can exacerbate interstitial lung disease and lead to disease progression. Notably, GERD remains relevant for postoperative management, as up to 75% of lung transplant patients may experience this [79, 80]. Chronic microaspiration of gastric fluids, a consequence of GERD, is a risk factor for bronchiolitis obliterans syndrome after initially successful lung transplantation [81]. Thus, RYGB emerges as a potentially preferred procedure in this patient population. In addition, RYBG after transplantation, particularly in scleroderma and/or interstitial lung disease recipient populations, may improve or stabilize long-term pulmonary function tests [78].

In conclusion, MBS holds promise as an adjunctive therapy for potential lung transplant candidates limited by their preoperative BMI, offering potential benefits in terms of improved eligibility, surgical feasibility, weight loss, and enhanced respiratory function. However, careful patient selection, timing, and comprehensive postoperative care are crucial for optimizing outcomes.

### MBS, transplant, and pharmaceutical considerations

In most cases of SOT, lifelong immunosuppression is essential for long-term graft survival. Immunosuppressant pharmacoavailability is highly dependent on gastrointestinal metabolism and absorption. MBS has the potential to significantly alter drug pharmacokinetics and absorption [82].

There is a paucity of robust research investigating the pharmacokinetics of immunosuppression in patients who have undergone bariatric surgery and SOT. A historic experience with open RYGB in renal transplant candidates demonstrated a significant increase in weight-adjusted dose requirements of cyclosporin after RYGB [83]. A small case series of 6 patients with RYGB receiving their first dose of tacrolimus, sirolimus, and mycophenolate showed differences in immunosuppressant pharmacokinetics post-MBS [84]. A larger study performed an immunosuppression drug analysis on 34 patients who had undergone both MBS and solid SOT (kidney, *N* = 26; liver/kidney, *N* = 1; kidney/pancreas, and liver, *N* = 4, and heart, *N* = 1) showed that post-MBS, 47% of the tacrolimus trough levels stayed within therapeutic values. The authors observed that the blood levels of tacrolimus declined slightly but remained within the therapeutic range. None of the patients required any significant tacrolimus dosage adjustment, nor was there any change in the mycophenolic acid or prednisone dosage [82]. In another study, renal transplant candidates who underwent SG did not appear to have significantly different pharmacokinetics of tacrolimus (immediate or extended-release) or mycophenolic acid, suggesting that post-SG patients may not require dose modification out of the norm for transplant recipients [85]. A prospective, single-dose pharmacokinetic study was performed prior to and after laparoscopic SG for tacrolimus, extended-release tacrolimus, mycophenolate mofetil, and enteric-coated mycophenolate sodium. Maximal concentrations appeared to have increased, and total clearance was decreased following laparoscopic SG [86]. Finally, a large single-center study looking at simultaneous LTx and sleeve gastrectomy used a standardized immunosuppression protocol that was the same for patients undergoing LTx or simultaneous LTx and SG. In that study, monitoring and immunosuppression management were not significantly different between those groups [42].

There exists the potential for altered pharmacokinetics in patients who have undergone both MBS and SOT. Currently available data suggests that dose modification does not appear to be profoundly different for patients who have not undergone MBS; however, appropriate monitoring is recommended for recipients of both SG and RYGB.

### MBS, transplant, and psychological considerations

The psychological and social factors of patients with end-stage organ disease play a crucial role in determining their candidacy, predicting posttransplant outcomes, and ensuring their ability to cope with the demands of transplantation [87]. Adherence to lifelong and lifesaving medication regimens (ie, immunosuppressants, antivirals, and others), abstinence from life-threatening behaviors (ie, alcohol consumption, smoking, and unhealthy diet), family support, and a strong therapeutic alliance with the treatment team have proven to be strong predictors of survival in high-risk transplant patients. Moreover, a comprehensive psychosocial evaluation allows health care professionals to tailor interventions and support services specific to individual needs, thus enhancing the chances of positive surgical outcomes [88].

The presence of obesity adds another level of complexity to this already challenging patient population due to the behavioral lifestyle changes necessary to maintain results after surgery [89], the possibility of engaging again in poor eating habits [90], the increase in alcohol-use disorder after MBS, and the high prevalence of depression-related disorders [91]. Indeed, there is strong evidence that individuals who undergo MBS are at an elevated risk of alcohol use, theoretically caused by the decrease in ghrelin levels after surgery, which stimulates the central nervous system’s reward-seeking regions. Unfortunately, alcohol use is a predictor of worse surgical outcomes after transplantation and therefore should be closely monitored and managed, particularly in patients with preoperative risk factors [92]. Lastly, the identification and treatment of preexisting psychopathology before surgery has a very limited value due to the clinical complexity of the underlying end-stage organ disease.

Preoperative psychosocial assessment involves the use of standardized tools, including structured interviews, self-report questionnaires, and clinician-administered scales. Commonly used assessment tools include the Psychosocial Assessment of Candidates for Transplantation, the Psychological Levels System, and the Transplant Evaluation Rating Scale [93, 94]. More recently, a new tool for the psychosocial evaluation of these patients has been developed, the Stanford Integrated Psychosocial Assessment for Transplantation [95]. Its advantages include the standardization of the evaluation process between patients as well as the ability to identify subjects who are at a higher risk of negative outcomes after transplant surgery. By assessing mental health, social support, adherence, and financial considerations, health care teams can develop individualized care plans that address the unique needs of each patient. Integrating psychosocial assessment into the pretransplant process fosters holistic patient care and supports successful long-term adaptation to the challenges of transplantation while setting reasonable expectations after both surgeries.

Although the Stanford Integrated Psychosocial Assessment for Transplantation was initially developed for use in screening potential candidates for organ transplantation, it has been suggested that this psychosocial assessment tool may have an important role in the context of bariatric patients [96]. Therefore, this tool may be used in future studies for the screening of patients for transplant and concomitant bariatric surgery.

## The obesity paradox

Despite the reported benefits of MBS in patients undergoing SOT, there remains a reluctance to refer patients for MBS due to various factors, including the obesity paradox. This phenomenon was initially observed in patients with ESKD awaiting kidney transplants and has also been noted with end-stage HF. Some studies describe paradoxical inverse associations between obesity and mortality, showcasing a 2-year survival rate of 68% for individuals with a BMI of ≥ 30 kg/m^2^, compared with 58% for those with a lower BMI. These outcomes have prompted transplant surgeons to reconsider providing weight loss interventions to patients with obesity, anticipating improved transplant outcomes. The protective effect of obesity against protein-energy malnutrition, which leads to decreased appetite, muscle loss, cardiovascular issues, and mortality, offers a potential explanation for this paradox. However, inconsistencies persist in the literature, with ongoing investigations into the precise mechanisms of the obesity paradox. Nevertheless, it remains crucial to offer preoperative counseling on an individualized basis, considering various patient- and surgery-related factors to ensure optimal outcomes [97, 98].

## A collaborative, multidisciplinary approach

Optimal management of obesity in patients with end-stage organ disease is a challenging task that requires an efficient collaborative effort and a multidisciplinary approach, including bariatric surgeons, transplant surgeons, and obesity and transplant medicine specialists. This specific patient population is subject to unique preoperative, intraoperative, and postoperative complications, which lead to higher morbidity and mortality. Therefore, the improvement and optimization of preexisting medical comorbidities by medical specialists leads to superior posttransplant surgical outcomes and long-term positive results [99]. A decision to proceed with MBS should involve all the implicated medical providers as the role of a multidisciplinary team remains primordial to obtain optimal outcomes.

### Limitations

Our review was subject to the inherent limitations of a literature review. The heterogeneity in the included papers may introduce some bias related to different study designs, sample sizes, follow-up periods, treatment modalities, and quality of research. Gaps in the current literature were filled with expert opinion and commentary from the authors of this manuscript, which might be a source of authority bias. Lastly, our review was focused only on surgical options for weight loss management.

## Conclusion

MBS for patients awaiting SOT is a complex procedure that requires careful consideration of physiologic, pharmacologic, and psychological components. Obesity and end-stage organ failure can occur concomitantly in the same patient, which reinforces the need for personalized care. A thorough multidisciplinary preoperative assessment is required to encourage clear discussion with the patient and determine personal long-term goals of therapy. A close postoperative evaluation is also necessary to adjust pharmacologic treatment, assess psychological status with validated tests, and make the required adjustments to obtain favorable long-term results. Further high-quality research in this area will allow for better recommendations and therefore treatment strategies for these complex patients. Ultimately, some combination of transplantation surgery and MBS may improve long-term outcomes for selected patients in terms of improved graft function, obesity management, and obesity-related medical conditions resolution.

### Supplementary Information

Below is the link to the electronic supplementary material.

Supplementary file1 (DOCX 15 KB)

## Data Availability

The study data is available from the corresponding author upon reasonable request.

## Abbreviations

ASTS: American Society for Transplant Surgery
BMI: Body mass index
CKD: Chronic kidney disease
ELD: End-stage liver disease
ESKD: End-stage kidney disease
GERD: Gastroesophageal reflux disease
HF: Heart failure
KTx: Kidney transplantation
LTx: Liver transplantation
LVAD: Left ventricular assist device
MASLD: Metabolic dysfunction-associated steatotic liver disease
MASH: Metabolic dysfunction-associated steatohepatitis
MBS: Metabolic and bariatric surgery
MELD: Model for end-stage liver disease
NASH: Nonalcoholic steatohepatitis
RYGB: Roux-en-Y gastric bypass
SAGES: Society of American Gastrointestinal and Endoscopic Surgeons
SG: Sleeve gastrectomy
SIPAT: Stanford Integrated Psychosocial Assessment for Transplantation
SOT: Solid organ transplantation
T2DM: Type-2 diabetes mellitus
US: United States
