# Correction: Obesity, organ failure, and transplantation: a review of the role of metabolic and bariatric surgery in transplant candidates and recipients

**DOI:** 10.1007/s00464-024-11043-y

**Published:** 2024-07-10

**Authors:** Omar M. Ghanem, Alejandro Pita, Mustafa Nazzal, Shaneeta Johnson, Tayyab Diwan, Nabeel R. Obeid, Kristopher P. Croome, Robert Lim, Cristiano Quintini, Bryan A. Whitson, Holly Ann Burt, Charles Miller, Matthew Kroh, SAGES & ASTS

**Affiliations:** 1 Department of Surgery, Mayo Clinic Rochester, Rochester, MN USA; 2 Department of Surgery, Cleveland Clinic Foundation, Cleveland, OH USA; 3 Department of Surgery, Saint Louis University Hospital, St. Louis, MO USA; 4 Department of Surgery, Morehouse School of Medicine, Atlanta, GA USA; 5 Department of Surgery, University of Michigan, Ann Arbor, MI USA; 6 Department of Transplant, Mayo Clinic Florida, Jacksonville, FL USA; 7 Atrium Health Carolinas Medical Center, Wake Forest University School of Medicine, Charlotte, NC USA; 8 Digestive Disease Institute, Cleveland Clinic Abu Dhabi, Abu Dhabi, United Arab Emirates; 9 Division of Cardiac Surgery, Department of Surgery, The Ohio State University Wexner Medical Center, Columbus, OH USA; 10 Society of American Gastrointestinal and Endoscopic Surgeons (SAGES), Los Angeles, CA USA

**Correction to: Surgical Endoscopy** 10.1007/s00464-024-10930-8

The original online version of this article was revised to correct the copyright information, which is as follows:

© The Author(s) 2024. Published by Elsevier Inc on behalf of the American Society of Transplantation and the American Society of Transplant Surgeons, and by Springer Nature on behalf of the Society of American Gastrointestinal and Endoscopic Surgeons (SAGES) and the European Association for Endoscopic Surgery (EAES).

The original article has been corrected.

