# Supplementary material for: Obesity, organ failure, and transplantation: a review of the role of metabolic and bariatric surgery in transplant candidates and recipients
Source: Surg Endosc. 2024 Jul 1;38(8):4138–51. doi: 10.1007/s00464-024-10930-8 (PMC11289013; doi:10.1007/s00464-024-10930-8)
Supplement: Supplementary file 1 — Supplementary file1 (DOCX 15 KB) [file 464_2024_10930_MOESM1_ESM.docx]

| **Authors** | **Type of study** | **Organ** | **Procedures** | **Sample Size** |
| --- | --- | --- | --- | --- |
| Elli EF et al. (29) | Retrospective Cohort | Kidney, Liver, Pancreas | SG | 10 (6 Kidney; 2 Liver; 2 Pancreas) |
| Khoraki J et al (35) | Retrospective Cohort | Kidney, Liver, Heart | SG | 10 (4 Kidney, 5 Liver, 1 Heart) |
| Chierici A et al (36) | Meta-analysis | Liver | SG, RYGB, AGB, DS, Jejuno-ileal bypass | 201 |
| Sharpton SR et al (41) | Retrospective Cohort | Liver | SG | 14 |
| Zamora-Valdes D et al (42) | Retrospective Cohort | Liver | SG | 29 |
| Tariq N et al (43) | Retrospective Cohort | Liver | SG | 42 |
| Fernando S et al (51) | Meta-analysis | Kidney | SG, RYGB, DS | 198 |
| Challapalli J et al (52) | Meta-analysis | Heart | SG | 59 |
| Sharma I et al (55) | Meta-analysis | Heart | SG, RYGB | 271 |
| Lee Y et al (61) | Meta-analysis | Heart | SG, RYGB, AGB | 98 |
| Chaudhry UI et al (62) | Retrospective Cohort | Heart | SG | 6 |
| Zenilman A et al (63) | Retrospective Cohort | Heart | SG | 6 |
| Punchai S et al (66) | Retrospective Cohort | Heart | SG | 17 |
| Lim CP et al (67) | Retrospective Cohort | Heart | SG, AGB | 7 |
| Ng M et al (68) | Retrospective Cohort | Heart | SG | 22 |
| Greene J et al (70) | Retrospective Cohort | Heart | SG | 3 |
| Orandi BJ et al (77) | Meta-analysis | Kidney, Liver, Lung, Heart | SG, RYGB | 439 (82 Heart, 28 Lung, 41 Liver, 288 Kidney) |
| Ardila-Gatas J et al (78) | Retrospective Cohort | Lung | RYGB, SG, AGB | 25 |
| Marterre WF et al (83) | Retrospective Cohort | Kidney | RYGB | 3 |

**Supplementary Table 1**. Articles summary
